# 4-Amino-2-methyl­quinoline monohydrate

**DOI:** 10.1107/S1600536808013093

**Published:** 2008-05-10

**Authors:** Xi-Shi Tai, Jun Xu, Yi-Min Feng, Zu-Pei Liang

**Affiliations:** a Department of Chemistry and Chemical Engineering, Weifang University, Weifang 261061, People’s Republic of China; b Weifang Institute of Supervision and Inspection of Product Quality, Weifang 261031, People’s Republic of China

## Abstract

The crystal structure of the title compound, C_10_H_10_N_2_·H_2_O, is stabilized by inter­molecular O—H⋯N, N—H⋯O and N—H⋯N hydrogen bonds.

## Related literature

For related literature, see: Tai *et al.* (2003 ▶, 2008 ▶); Tai, Yin & Feng (2007 ▶); Tai, Yin & Hao (2007 ▶); Tai, Yin *et al.* (2007 ▶ ▶); Tai & Feng (2008 ▶); Wang *et al.* (2007 ▶).
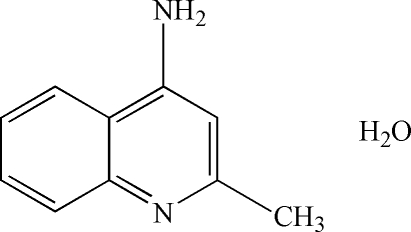


## Experimental

### 

#### Crystal data

C_10_H_10_N_2_·H_2_O
*M* *_r_* = 176.22
Orthorhombic, 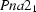

*a* = 4.7432 (8) Å
*b* = 13.9070 (13) Å
*c* = 14.5129 (16) Å
*V* = 957.3 (2) Å^3^
*Z* = 4
Mo *K*α radiation
μ = 0.08 mm^−1^
*T* = 298 (2) K
0.43 × 0.35 × 0.32 mm

#### Data collection

Bruker SMART CCD area-detector diffractometer
Absorption correction: multi-scan (*SADABS*; Bruker, 2000 ▶) *T*_min_ = 0.966, *T*_max_ = 0.975
3925 measured reflections
882 independent reflections
716 reflections with *I* > 2σ(*I*)
*R*_int_ = 0.029

#### Refinement

*R*[*F* ^2^ > 2σ(*F* ^2^)] = 0.032
*wR*(*F* ^2^) = 0.093
*S* = 1.04
882 reflections
119 parameters
1 restraint
H-atom parameters constrained
Δρ_max_ = 0.10 e Å^−3^
Δρ_min_ = −0.11 e Å^−3^

### 

Data collection: *SMART* (Bruker, 2000 ▶); cell refinement: *SAINT* (Bruker, 2000 ▶); data reduction: *SAINT*; program(s) used to solve structure: *SHELXS97* (Sheldrick, 2008 ▶); program(s) used to refine structure: *SHELXL97* (Sheldrick, 2008 ▶); molecular graphics: *SHELXTL* (Sheldrick, 2008 ▶); software used to prepare material for publication: *SHELXTL*.

## Supplementary Material

Crystal structure: contains datablocks global, I. DOI: 10.1107/S1600536808013093/at2564sup1.cif

Structure factors: contains datablocks I. DOI: 10.1107/S1600536808013093/at2564Isup2.hkl

Additional supplementary materials:  crystallographic information; 3D view; checkCIF report

## Figures and Tables

**Table 1:** Hydrogen-bond geometry (Å, °)

*D*—H⋯*A*	*D*—H	H⋯*A*	*D*⋯*A*	*D*—H⋯*A*
O1—H1⋯O1^i^	0.85	1.94	2.791 (3)	174
O1—H2⋯N1^ii^	0.85	1.96	2.805 (3)	171
N2—H2*A*⋯O1^iii^	0.86	2.10	2.947 (4)	168
N2—H2*B*⋯N2^iv^	0.86	2.51	3.321 (4)	158

Symmetry codes: (i) 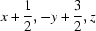; (ii) 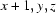; (iii) 
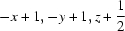
; (iv) 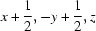.

**Fig. 1.**
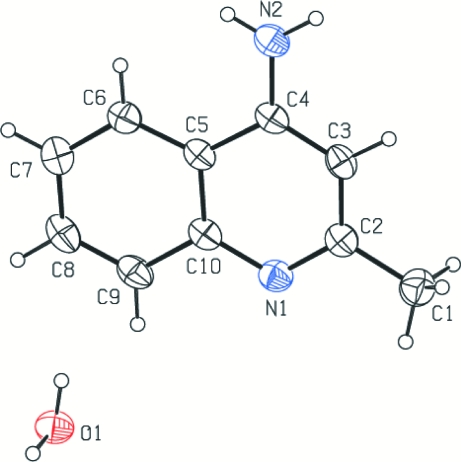
The molecular structure of (I) showing 30% displacement ellipsoids.

## supplementary crystallographic information

### Comment

As part of our ongoing studies of the coordination chemistry of ligands containing nitrogen (Tai *et al.*, 2003; Tai, Yin & Feng, 2007; Tai, Yin, Feng & Kong, 2007; Tai, Yin & Hao, 2007; Tai & Feng, 2008; Tai, Feng & Zhang, 2008; Wang *et al.*, 2007), we now report the structure of the title compound, (I), (Fig. 1).

In the molecule of (I), the geometrical parameters for (I) are normal. The packing is stabilized by the intermolecular O—H···N, N—H···O and N—H···N hydrogen bonds (Table 1).

### Experimental

1 mmol of Ethyl benzoylacetate was added to a solution of 4-amino-2-methylquinoline (1 mmol) in 10 ml of 95% ethanol. The mixture was stirred for 2 h at refluxing temperature. Evaporating some ethanol, clear blocks of (I) were obtained after one weeks.

### Refinement

The H atoms were placed geometrically (C—H = 0.93–0.96 Å, O—H = 0.852 Å, N—H = 0.86 Å) and refined as riding with *U*_iso_(H) = 1.2 or 1.5*U*_eq_(carrier).

### Crystal data

**Table:**

C_10_H_10_N_2_·H_2_O_1_	*F*_000_ = 376
*M**_r_* = 176.22	*D*_x_ = 1.223 Mg m^−^^3^
Orthorhombic, *P**n**a*2_1_	Mo *K*α radiation λ = 0.71073 Å
Hall symbol: P 2c -2n	Cell parameters from 1365 reflections
*a* = 4.7432 (8) Å	θ = 2.8–23.5º
*b* = 13.9070 (13) Å	µ = 0.08 mm^−^^1^
*c* = 14.5129 (16) Å	*T* = 298 (2) K
*V* = 957.3 (2) Å^3^	Block, colourless
*Z* = 4	0.43 × 0.35 × 0.32 mm

### Data collection

**Table:**

Bruker SMART CCD area-detector diffractometer	882 independent reflections
Radiation source: fine-focus sealed tube	716 reflections with *I* > 2σ(*I*)
Monochromator: graphite	*R*_int_ = 0.029
*T* = 298(2) K	θ_max_ = 25.0º
φ and ω scans	θ_min_ = 2.0º
Absorption correction: multi-scan(SADABS; Bruker, 2000)	*h* = −5→5
*T*_min_ = 0.966, *T*_max_ = 0.975	*k* = −16→15
3925 measured reflections	*l* = −14→17

### Refinement

**Table:**

Refinement on *F*^2^	Secondary atom site location: difference Fourier map
Least-squares matrix: full	Hydrogen site location: inferred from neighbouring sites
*R*[*F*^2^ > 2σ(*F*^2^)] = 0.032	H-atom parameters constrained
*wR*(*F*^2^) = 0.093	*w* = 1/[σ^2^(*F*_o_^2^) + (0.0498*P*)^2^ + 0.1333*P*] where *P* = (*F*_o_^2^ + 2*F*_c_^2^)/3
*S* = 1.05	(Δ/σ)_max_ < 0.001
882 reflections	Δρ_max_ = 0.10 e Å^−^^3^
119 parameters	Δρ_min_ = −0.11 e Å^−^^3^
1 restraint	Extinction correction: none
Primary atom site location: structure-invariant direct methods	

### Special details

**Table:**

Geometry. All e.s.d.'s (except the e.s.d. in the dihedral angle between two l.s. planes) are estimated using the full covariance matrix. The cell e.s.d.'s are taken into account individually in the estimation of e.s.d.'s in distances, angles and torsion angles; correlations between e.s.d.'s in cell parameters are only used when they are defined by crystal symmetry. An approximate (isotropic) treatment of cell e.s.d.'s is used for estimating e.s.d.'s involving l.s. planes.
Refinement. Refinement of *F*^2^ against ALL reflections. The weighted *R*-factor *wR* and goodness of fit *S* are based on *F*^2^, conventional *R*-factors *R* are based on *F*, with *F* set to zero for negative *F*^2^. The threshold expression of *F*^2^ > σ(*F*^2^) is used only for calculating *R*-factors(gt) *etc*. and is not relevant to the choice of reflections for refinement. *R*-factors based on *F*^2^ are statistically about twice as large as those based on *F*, and *R*- factors based on ALL data will be even larger.

### Fractional atomic coordinates and isotropic or equivalent isotropic displacement parameters (Å^2^)

**Table:**

	*x*	*y*	*z*	*U*_iso_*/*U*_eq_	
N1	0.0728 (5)	0.56239 (17)	0.32460 (17)	0.0508 (7)	
N2	0.2819 (6)	0.33360 (19)	0.49988 (18)	0.0612 (7)	
H2A	0.1996	0.3288	0.5525	0.073*	
H2B	0.4034	0.2911	0.4832	0.073*	
O1	0.9245 (4)	0.69712 (15)	0.18915 (16)	0.0582 (6)	
H1	1.0702	0.7326	0.1914	0.070*	
H2	0.9510	0.6552	0.2309	0.070*	
C1	−0.2458 (8)	0.6300 (2)	0.4362 (3)	0.0672 (9)	
H1A	−0.2491	0.6802	0.3907	0.101*	
H1B	−0.1891	0.6563	0.4945	0.101*	
H1C	−0.4306	0.6024	0.4417	0.101*	
C2	−0.0410 (6)	0.5538 (2)	0.4074 (2)	0.0488 (8)	
C3	0.0273 (7)	0.4782 (2)	0.46651 (19)	0.0485 (7)	
H3	−0.0600	0.4746	0.5238	0.058*	
C4	0.2193 (6)	0.4091 (2)	0.44254 (18)	0.0452 (7)	
C5	0.3470 (6)	0.4156 (2)	0.35341 (19)	0.0429 (7)	
C6	0.5474 (7)	0.3508 (2)	0.3196 (2)	0.0523 (8)	
H6	0.6030	0.2992	0.3562	0.063*	
C7	0.6634 (8)	0.3615 (3)	0.2340 (2)	0.0608 (9)	
H7	0.7952	0.3175	0.2124	0.073*	
C8	0.5828 (7)	0.4388 (3)	0.1796 (3)	0.0631 (9)	
H8	0.6624	0.4464	0.1215	0.076*	
C9	0.3892 (7)	0.5037 (2)	0.20989 (19)	0.0568 (9)	
H9	0.3382	0.5549	0.1722	0.068*	
C10	0.2654 (6)	0.4944 (2)	0.29716 (19)	0.0455 (7)	

### Atomic displacement parameters (Å^2^)

**Table:**

	*U*^11^	*U*^22^	*U*^33^	*U*^12^	*U*^13^	*U*^23^
N1	0.0487 (14)	0.0540 (14)	0.0498 (15)	−0.0009 (12)	−0.0061 (12)	0.0071 (12)
N2	0.0754 (19)	0.0656 (16)	0.0428 (14)	0.0058 (15)	0.0043 (13)	0.0133 (12)
O1	0.0614 (12)	0.0590 (11)	0.0544 (12)	−0.0011 (11)	−0.0117 (12)	0.0083 (10)
C1	0.062 (2)	0.066 (2)	0.074 (2)	0.0027 (18)	0.0037 (19)	−0.0011 (17)
C2	0.0423 (17)	0.0539 (17)	0.0502 (18)	−0.0078 (14)	−0.0049 (14)	−0.0016 (15)
C3	0.0465 (16)	0.0590 (18)	0.0401 (16)	−0.0100 (15)	0.0010 (13)	−0.0024 (14)
C4	0.0428 (17)	0.0520 (16)	0.0406 (15)	−0.0113 (14)	−0.0062 (13)	0.0053 (13)
C5	0.0404 (15)	0.0502 (16)	0.0381 (14)	−0.0100 (13)	−0.0040 (12)	0.0022 (11)
C6	0.0500 (17)	0.0549 (17)	0.0519 (18)	−0.0033 (14)	−0.0021 (15)	0.0059 (14)
C7	0.056 (2)	0.070 (2)	0.056 (2)	−0.0027 (16)	0.0065 (17)	−0.0026 (16)
C8	0.059 (2)	0.084 (2)	0.0464 (16)	−0.0094 (18)	0.0060 (17)	0.0093 (18)
C9	0.0569 (18)	0.069 (2)	0.0440 (19)	−0.0077 (18)	−0.0041 (14)	0.0147 (14)
C10	0.0416 (15)	0.0540 (17)	0.0408 (15)	−0.0100 (13)	−0.0067 (13)	0.0042 (13)

### Geometric parameters (Å, °)

**Table:**

N1—C2	1.322 (4)	C3—H3	0.9300
N1—C10	1.374 (4)	C4—C5	1.431 (4)
N2—C4	1.372 (4)	C5—C6	1.399 (4)
N2—H2A	0.8600	C5—C10	1.420 (4)
N2—H2B	0.8600	C6—C7	1.367 (5)
O1—H1	0.8500	C6—H6	0.9300
O1—H2	0.8499	C7—C8	1.387 (5)
C1—C2	1.497 (5)	C7—H7	0.9300
C1—H1A	0.9600	C8—C9	1.361 (5)
C1—H1B	0.9600	C8—H8	0.9300
C1—H1C	0.9600	C9—C10	1.402 (4)
C2—C3	1.396 (4)	C9—H9	0.9300
C3—C4	1.369 (4)		
			
C2—N1—C10	118.2 (2)	N2—C4—C5	120.3 (3)
C4—N2—H2A	120.0	C6—C5—C10	118.7 (3)
C4—N2—H2B	120.0	C6—C5—C4	124.3 (3)
H2A—N2—H2B	120.0	C10—C5—C4	116.9 (3)
H1—O1—H2	104.5	C7—C6—C5	121.4 (3)
C2—C1—H1A	109.5	C7—C6—H6	119.3
C2—C1—H1B	109.5	C5—C6—H6	119.3
H1A—C1—H1B	109.5	C6—C7—C8	119.4 (3)
C2—C1—H1C	109.5	C6—C7—H7	120.3
H1A—C1—H1C	109.5	C8—C7—H7	120.3
H1B—C1—H1C	109.5	C9—C8—C7	121.1 (3)
N1—C2—C3	122.1 (3)	C9—C8—H8	119.4
N1—C2—C1	117.0 (3)	C7—C8—H8	119.4
C3—C2—C1	120.8 (3)	C8—C9—C10	120.8 (3)
C4—C3—C2	121.8 (3)	C8—C9—H9	119.6
C4—C3—H3	119.1	C10—C9—H9	119.6
C2—C3—H3	119.1	N1—C10—C9	118.4 (3)
C3—C4—N2	121.8 (3)	N1—C10—C5	123.1 (3)
C3—C4—C5	117.9 (3)	C9—C10—C5	118.5 (3)
			
C10—N1—C2—C3	−0.4 (4)	C5—C6—C7—C8	0.6 (5)
C10—N1—C2—C1	178.8 (3)	C6—C7—C8—C9	−0.4 (5)
N1—C2—C3—C4	0.9 (4)	C7—C8—C9—C10	0.1 (5)
C1—C2—C3—C4	−178.3 (3)	C2—N1—C10—C9	−179.3 (2)
C2—C3—C4—N2	−178.6 (3)	C2—N1—C10—C5	0.1 (4)
C2—C3—C4—C5	−1.0 (4)	C8—C9—C10—N1	179.5 (3)
C3—C4—C5—C6	179.6 (3)	C8—C9—C10—C5	0.1 (4)
N2—C4—C5—C6	−2.8 (4)	C6—C5—C10—N1	−179.2 (3)
C3—C4—C5—C10	0.7 (4)	C4—C5—C10—N1	−0.2 (4)
N2—C4—C5—C10	178.3 (2)	C6—C5—C10—C9	0.1 (4)
C10—C5—C6—C7	−0.5 (4)	C4—C5—C10—C9	179.1 (2)
C4—C5—C6—C7	−179.4 (3)		

### Hydrogen-bond geometry (Å, °)

**Table:**

*D*—H···*A*	*D*—H	H···*A*	*D*···*A*	*D*—H···*A*
O1—H1···O1^i^	0.85	1.94	2.791 (3)	174
O1—H2···N1^ii^	0.85	1.96	2.805 (3)	171
N2—H2A···O1^iii^	0.86	2.10	2.947 (4)	168
N2—H2B···N2^iv^	0.86	2.51	3.321 (4)	158

Symmetry codes: (i) *x*+1/2, −*y*+3/2, *z*; (ii) *x*+1, *y*, *z*; (iii) −*x*+1, −*y*+1, *z*+1/2; (iv) *x*+1/2, −*y*+1/2, *z*.
